# Utilizing deep learning model for assessing melanocytic density in resection margins of lentigo maligna

**DOI:** 10.1186/s13000-024-01532-y

**Published:** 2024-08-03

**Authors:** Jan Siarov, Darshan Kumar, John Paoli, Johan Mölne, Martin Gillstedt, Noora Neittaanmäki

**Affiliations:** 1 Department of Laboratory Medicine, Institute of Biomedicine, Sahlgrenska Academy, University of Gothenburg, Gothenburg, Sweden; 2 Department of Clinical Pathology, Region Västra Götaland, Sahlgrenska University Hospital, Gothenburg, Sweden; 3 Aiforia Technologies Plc, Helsinki, Finland; 4 Department of Dermatology, Institute of Clinical Sciences, Sahlgrenska Academy, University of Gothenburg, Gothenburg, Sweden; 5 Department of Dermatology and Venereology, Region Västra Götaland, Sahlgrenska University Hospital, Gothenburg, Sweden

**Keywords:** Deep learning, Computational pathology, Lentigo maligna, Margin assessment, Melanocytic count

## Abstract

**Background:**

Surgical excision with clear histopathological margins is the preferred treatment to prevent progression of lentigo maligna (LM) to invasive melanoma. However, the assessment of resection margins on sun-damaged skin is challenging. We developed a deep learning model for detection of melanocytes in resection margins of LM.

**Methods:**

In total, 353 whole slide images (WSIs) were included. 295 WSIs were used for training and 58 for validation and testing. The algorithm was trained with 3,973 manual pixel-wise annotations. The AI analyses were compared to those of three blinded dermatopathologists and two pathology residents, who performed their evaluations without AI and AI-assisted. Immunohistochemistry (SOX10) served as the reference standard. We used a dichotomized cutoff for low and high risk of recurrence (≤ 25 melanocytes in an area of 0.5 mm for low risk and > 25 for high risk).

**Results:**

The AI model achieved an area under the receiver operating characteristic curve (AUC) of 0.84 in discriminating margins with low and high recurrence risk. In comparison, the AUC for dermatopathologists ranged from 0.72 to 0.90 and for the residents in pathology, 0.68 to 0.80. Additionally, with aid of the AI model the performance of two pathologists significantly improved.

**Conclusions:**

The deep learning showed notable accuracy in detecting resection margins of LM with a high versus low risk of recurrence. Furthermore, the use of AI improved the performance of 2/5 pathologists. This automated tool could aid pathologists in the assessment or pre-screening of LM margins.

**Supplementary Information:**

The online version contains supplementary material available at 10.1186/s13000-024-01532-y.

## Background

Melanomas arising in extensively sun-damaged skin, identified by pronounced solar elastosis, are categorized as high chronic sun-damaged (high-CSD) melanomas. These tumors demonstrate distinctive clinical and genetic characteristics. Lentigo maligna melanoma (LMM), a variant of high-CSD melanoma, has a precursor lesion referred to as lentigo maligna (LM). This in situ variant is distinguished by the lentiginous proliferation of predominantly individual cytologically atypical melanocytes within the basal epidermis [1].

Precise evaluation of residual tumor presence within resection margins is crucial in both the assessment and subsequent treatment of patients afflicted with LM and LMM [2]. Distinguishing between LM and sun-induced melanocytic hyperplasia also poses a significant challenge for pathologists involved in assessing the completeness of resection margins. The subtle features of LM can be difficult to identify, in particular at the edges of the lesion where histopathologic features may resemble those found in unaffected, sun-damaged skin [3, 4]. One of the key criteria utilized for this differentiation is the increased number of junctional melanocytes observed in hematoxylin and eosin- (H&E) stained sections in combination with histological features including melanocytic nests, irregular distribution of melanocytes, descent of melanocytes far down adnexal epithelial structures and melanocytic pleomorphism [5, 6]. Given the time-consuming and challenging process of evaluation of resection margins, it is evident that new solutions are needed.

Several published studies have identified a greater average melanocytic density in LM compared to sun-induced melanocytic hyperplasia [7, 8]. Immunohistochemistry (IHC) can be used to facilitate the evaluation of resection margins [9, 10]. SOX10-staining increases the observed melanocytic density (MD) as compared to H&E-staining when evaluating the surgical margins of LM [11]. Furthermore, the utilization of SOX10 staining notably reduces interobserver variability among pathologists [12]. Previously MD of ≥ 25 melanocytes in a 0.5-mm long field at the resection margin has shown to be a robust predictor of LM recurrence [8].

Employing digital whole slide imaging (WSI) facilitates the integration of artificial intelligence (AI) into digital pathology [13]. This integration provides a potential to increase diagnostic precision, while also to reduce the time to diagnosis and the interobserver variability [14–16]. However, there are only a few studies regarding the use of AI in assessment of histopathological resection margins [17]. Deep learning models are widely applied on melanoma diagnostics [14]. However, there are no previous publications to our knowledge regarding deep learning methods in assessment of the LM resection margins.

The aim of this study was to investigate the feasibility of a deep learning algorithm for automated evaluation of MD in resection margins of LM and assessment of the recurrence risk. Additionally, we aimed to assess whether the performance of individual pathologists could improve with the assistance of AI.

## Methods

### Dataset

The inclusion criteria were LM excised and histopathologically verified at the Department of Pathology at Sahlgrenska University Hospital between January 1, 2020 and May 10, 2023 with a representative haematoxyllin-eosin (H&E)-stained glass slide from formalin-fixed and paraffin-embedded tissue available for scanning. In total, 355 cases fulfilled these criteria. Cases with both positive and negative margins were collected. A single slide per case containing either the closest surgical margin or harboring the highest MD at the margin was included in the study. This was evaluated by an experienced dermatopathologist (JS). The glass slides were anonymized and then digitally scanned for WSI with a 40 × mode Nanozoomer S210 Digital Slide Scanner (Hamamatsu Photonics K.K., Shizuoka, Japan) with a resolution of 0.23 µm/pixel and using a 20 × objective lens. After scanning, two H&E WSIs needed to be excluded due to poor image quality.

Thus, the dataset included 353 H&E-stained WSIs. However, the developed AI model was trained and tested on smaller regions selected on these WSIs. The training set consisted of 729 regions within 295 WSIs. The remaining 58 WSIs were used to define an additional 88 regions, with 33 used for validation and 58 for testing, (Table 1).

**Table 1:** The number of included lentigo maligna whole slide images included in the training and the validation and test set and their corresponding training, validation and test regions

	H&E WSIs	Regions
Training set	295*	729 training regions
Test and validation set	58**	30 validation regions 58 test regions
Total	353	817 regions

*65 with available SOX10

**SOX10 available for all cases

*WSI* whole slide image

*H&E*  hematoxylin and eosin

### Immunostainings and ground truth

In cases where additional SOX10-stained slides were available in the archives (*n* = 65), these were scanned along with the H&E slides. These cases were included in the training set in order to facilitate the annotations. Furthermore, for all 58 WSIs included in the test and validation set, new serial sections were acquired. The first section was stained with H&E and the following consecutive Sect. (4 μm between each slide) was stained with SOX10 (AVI 3099G, Ready-to-use, Clone BC34, Mouse Monoclonal Primary Antibody, BioCare Pacheco, CA, USA). Automated immunohistochemistry was performed on an AutoStainer Link-instrument (Agilent Dako, Copenhagen, Denmark) using the EnVision Flex K8000 (Agilent Dako, Copenhagen, Denmark), a high pH detection kit. Thus, 123 SOX10 WSIs (65 SOX10 slides for the training set and an additional 58 SOX10 slides for the validation and test set) were available to facilitate the annotations and to serve as ground truth for testing and validating. The MD on SOX10 slides served as the ground truth.

### Annotations

The training annotations were performed only on the training set while the test and validation WSIs were kept unannotated. The annotations were done manually by an experienced dermatopathologist with help of SOX10 when this was available (*n* = 65). In total, 3,973 melanocytes were manually annotated with a chosen diameter size of 10 μm and regardless of cellular atypia. Both lentiginous and nested growth patterns were annotated.

### Training the AI model

The AI model was generated using Aiforia Create Version 5.3 (Aiforia Technologies Plc, Helsinki, Finland), a commercial image management and analysis cloud platform which aids the development of a machine learning model with a convolutional neural network (CNN) and supervised learning. The annotations were provided as pixel-level segments. The trained CNNs produced semantic segmentation at pixel level meaning that each pixel is predicted to belong to either the foreground or background class. The segmentation-based outcome prediction was done using semantic segmentation. Here, a fully supervised pixel-level input was provided by the expert pathologist with help of IHC. The algorithm was trained to detect melanocytes according to the annotations.

To create the final AI model, a total of eleven training sessions were performed. After each training, additional annotations in the training set WSIs were made. Depending on the layer complexity, the training was programmed to prematurely terminate if the learning curve was not steep enough. This could directly be seen by the training loss function. Hence, a lower error percentage could be expected if the training loss was minimal after all the training rounds were reached, which is generally defined by a non-significant increase in the training curve. The final training was performed for 7,000 iterations out of which 4,452 iterations were executed (training loss 0.71). All 729 training regions were used, and verifications were carried out to yield an error of 8.6% (F1 Score; 95.6%). Patches that were used in training and inference were extracted from the image pyramid at a level that is dependent on the field of view (region layer) or object size (object layer). Morphometric analysis was enabled for the AI model prior to the image analysis run. Image analysis was performed in the Aiforia Hub for WSIs in multiple batches. Data was visually monitored, and the pixel-level segmentation outcome was generated using Microsoft Excel (Microsoft Corporation, Redmond, Washington, USA). The patch level information was extrapolated to slide level with the help of aggregated pixel-level segmentation outcomes.

### Validating the AI model

The performance of the AI model was validated on unannotated areas of WSIs using a pixel-wise validation. The validation of the potential performance of the algorithm was done prior to running the analyses on the test set. A total of 33 regions were used. These regions were manually drawn and were 0.5 mm in length and, similar to the ones in the test set, these only included the epidermal surface. The regions were unique for the validation and were not used in the training or test sets. The validation was done by comparing the AI analyses on the validation region to MD assessed by one dermatopathologist and compared to the ground truth (MD on SOX10-stained validation regions).

### Testing the AI model

The performance of the algorithm was tested against five pathologists as described below against the ground truth (MD on SOX10-stained test regions). An area measuring 0.5 mm in length from the resection margin was superimposed on the image at the surgical margin***.*** The subepidermal tissue was excluded since the analyzed region only covered the epidermis including adnexal structures.

Similar regions were digitally drawn on consecutive SOX10-stained slides. The MD on these regions were used as ground truth. Using the recurrence prediction model by Gorman et al [8], each region in the test set was labeled as low-risk or high-risk. A low-risk region was defined as having a MD of ≤ 25 melanocytes in an area of 0.5 mm in the resection margin and a high-risk region was defined as having > 25 melanocytes. A false negative result was defined as not being able to identify high-risk regions and a false positive result was defined as wrongly labeling a low-risk region as high-risk.

### Assessment by the pathologists

Three dermatopathologists (two with over 10 years of experience, considered senior dermatopathologists, and one with 2 years of experience, considered a junior dermatopathologist) independently counted the melanocytes on the test regions. They were blinded to the AI results, each other’s evaluations, and the original pathology reports. The pathologists could use variable digital magnifications to evaluate the slides and only had access to the H&E slides but didn’t have access to the SOX10 slides. There were no time constraints, and the results were reported separately for each region. This initial assessment was defined as round 1.

**Fig. 1:**
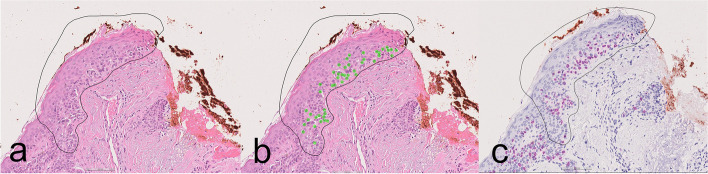
Representative images from one of the test regions with the black circle marking the area to be assessed: **a** H&E-stained slide without AI assistance, **b** H&E-stained slide with AI assistance showing green overlaid dots where the model identified melanocytes, and (**c**) SOX10 stain used as ground truth and not available for the evaluators

After a washout period of at least two weeks, the procedure was repeated (round 2) with the pathologists aided by the AI algorithm. Within each region, a green visualization overlay depicted the cells identified as positive by the AI, (Fig. 1). While the analysis tool could be hidden, the pathologists had the option to disregard this supplementary information or integrate it into their melanocyte counting process.

### Statistical analysis

No prior in-house data were available regarding the performance of an AI algorithm for this task. Therefore, no power analysis was used to predetermine an appropriate sample size. All data were analyzed using R version 3.5.3 (https://www.r-project.org/) together with a trained statistician.

The gain parameter regulates the CNN's sensitivity in identifying pixels associated with a specific feature class. Augmenting the gain enhances the CNN's capacity to identify pixels within the designated feature class. A receiver operating characteristic (ROC) curve was generated by using a cutoff of 26 melanocytes for both the true label and all the gain settings. Each point on this ROC curve therefore represents one gain setting. One ROC curve for each gain setting was also generated, treating the true label in the same way as above (cutoff at 26 melanocytes) but where the AI outcome for each gain is treated as a latent continuous variable, varying the threshold over all available values, generating an ROC curve and an area under the ROC (AUC) for each gain setting (seven different gains). Also, the pathologists’ ROC curves were generated in the same way. When comparing two ROC curves, DeLong’s paired test was used. All statistical tests were two-sided and *P* < 0.05 was considered statistically significant. Fleiss kappa was used to compare the inter-pathologist agreement.

## Results

### The recurrence risk assessment

Of the 58 regions included in the test set, 22 (37%) harbored > 25 melanocytes on the reference SOX10 regions and defined as high-risk margins while 36/58 (62%) harbored < 25 melanocytes and were defined as low-risk resection margins.

### Round 1 without the aid of AI

**Fig. 2:**
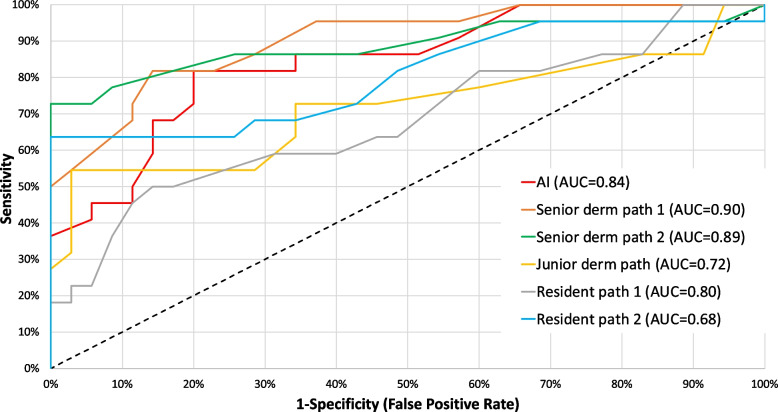
The receiver operating characteristic curves and corresponding area under these curves for the AI model and the individual dermatopathologists and pathology residents

The AI model’s performance in detection of high-risk resection margins demonstrated an AUC of 0.84 (95% CI: 0.73–0.96), (Fig. 2). For comparison, the AUCs for the three dermatopathologists were 0.72 (95% CI: 0.58–0.86), 0.89 (95% CI: 0.79–0.99) and 0.90 (95% CI: 0.81–0.99), while the AUCs for the two residents were 0.68 (95% CI: 0.54–0.83) and 0.80 (95% CI: 0.68–0.93).

The sensitivity for predicting high-risk regions was 45% (95% CI: 24–68%), 46% (95% CI: 24–68%) and 54% (95% CI: 32–76%) for the respective dermatopathologists and 36% (95% CI: 17–59%) and 64% (95% CI: 41–83%) for the residents compared to the AI’s sensitivity of 81% (95% CI: 60–95%). The specificity was 100% (95% CI: 90–100%), 100% (95% CI: 90–100%) and 97% (95% CI: 85–100%) for the dermatopathologists and 91% (95% CI: 77–98%) and 100% (95% CI: 90–100%) for the residents compared to 71% for the AI (95% CI: 54–85%).

As a result, in the first round, the AI outperformed the junior dermatopathologist (*p* = 0.004) and one resident (*p* = 0.01). There was no statistically significant difference between the AI and the other pathologists (*p*-values ranging between 0.14 and 0.43).

### Round 2 with the aid of AI

**Fig. 3:**
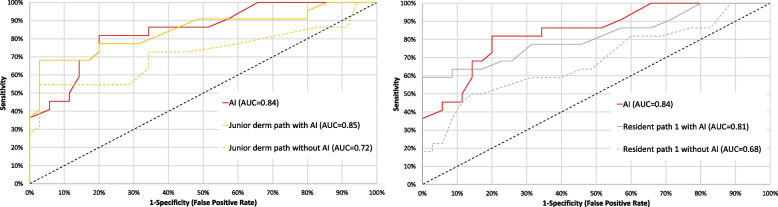
The receiver operating characteristic curves and corresponding area under these curves for the junior dermatopathologist (left) and one of the residents in pathology (right) with and without the aid of AI compared to the AI model (red curve)

In the second round, when the pathologists were aided by the AI, the performance of the junior dermatopathologist and one of the residents in pathology was significantly improved in comparison to round 1, (Fig. 3) and their AUCs increased from 0.84 (95% CI: 0.73–0.96) to 0.93 (95% CI: 0.85–1.00, *p* = 0.029) and from 0.68 (95% CI: 0.54–0.83) to 0.81 (95% CI: 0.69–0.94, *p *= 0.0057).

Furthermore, with the aid of the AI, the two senior dermatopathologists, performed significantly better than the AI model itself (Additional files Fig. 1) and their corresponding AUCs increased from 0.89 to 0.93 (*p* = 0.042, 95% CI: 0.85–1.00) and from 0.90 to 0.93 (95% CI: 0.85–1.00, *p* = 0.033).

The interobserver agreement for all five pathologists was 0.62 (95% CI: 0.54–0.70) in the first round which is generally interpreted as "substantial agreement”. With the integration of AI in round two, it increased to 0.69 (95% CI: 0.60–0.77, *p* = 0.63). Examples of true and false positive labeled regions are shown in the additional files, (Additional files Fig. 2).

## Discussion

The deep learning model showed high accuracy in assessment of the MD and, thus, in detecting high-risk versus low-risk LM resection margins. Interestingly the use of AI improved the performance of some pathologists. Precise evaluation of residual tumor presence within resection margins is crucial in both the assessment and subsequent treatment of patients afflicted with LM**.** The utilization of such an automated tool could aid pathologists in the assessment and/or pre-screening of LM excisions.

Interestingly, all pathologists showed a trend of increased performance when aided by the AI algorithm and this was statistically significant for the junior dermatopathologist and one of the pathology residents.

It is even likely that in a clinical environment the performance of the pathologists might be poorer and that they can get additional aid from the AI model. Assessment of multiple resection margins on multiple slides becomes a cumbersome task for the pathologist often requiring additional staining with IHC in ambiguous cases. Our model could potentially provide helpful aid in pre-screening the entirety of a scanned WSI, highlighting high-risk areas that need more urgent attention or identifying which slides need additional IHC prior to the case being assigned to a pathologist.

Three of the pathologists were trained dermatopathologists who regularly assess melanocytic lesions in their daily practice and are familiar with digital diagnostics. This tool may prove to be particularly advantageous in settings where specialized and experienced pathologists are not readily available, which was supported by the significantly improved performance of the junior dermatopathologist and one of the pathology residents.

High discordance in assessing the histopathological margins may lead to both over- and undertreatment of patients with LM [18]. The goal of therapy is to achieve complete removal of the LM in order to prevent development of invasive disease and surgical excision is therefore the treatment of choice to achieve clear histopathological margins. Although without statistical significance, the pathologists increased their interobserver agreement as a group with the aid of AI.

Distinguishing melanocytes from pigmented and non-pigmented basal keratinocytes and inflammatory cells can be challenging on routine H&E slides without the aid of IHC [9, 10].

The need for IHC makes interpretation of LM time-consuming and costly. The AI model was trained on annotated H&E slides with the aid of SOX10 staining, but the testing was performed solely on H&E slides. A notable strength of this study is that both the pathologists and the AI model assessed the same H&E-stained WSIs. Employing the AI model in a screening environment could reduce the need for IHC, reducing costs and shortening lead times in the laboratory.

We chose to employ the SOX10 immunostain because it's the reference standard at our department, showing nearly 100% sensitivity in detection of melanocytes in the epidermis [19].

Previous studies have shown that Melan-A staining can lead to an overestimation of melanocyte numbers in sun-damaged skin [20]. In contrast, SOX10 has proven to be a highly specific melanocytic marker that is not expressed in keratinocytes or lymphocytes. Another option is using PRAME, an emerging marker that has demonstrated high sensitivity and specificity for diagnosing LM in biopsies and excisions [21].

MD has been used in determining histopathological margins and predicting the risk of recurrence. A systematic review assessed the MD in LM and chronic sun-damaged skin [22]. In each individual study, mean MD scores were higher for LM than for chronic sun-damaged skin. However, upon examining the overall study situation, it becomes evident that data are highly diverse, and exhibit overlaps. Consequently, they came to the conclusion that it is not possible to determine a precise reference point. Particularly concerning the significance of defining surgical resection margins, this underscores the necessity for additional research to prevent patients from undergoing extensive and potentially disfiguring surgery without increasing recurrence risk for LM patients.

Gorman et al. demonstrated that an MD of > 25 melanocytes/0.5 mm field diameter at the resection margin serves as a robust predictor of LM recurrence [8]. They utilized both a two and a three-graded recurrence risk model (low, intermediate, and high-risk) but despite the risk of false negatives, we opted for their dichotomized low- and high-risk model for this initial pilot study. This division of MD into two risk strata yielded 95% sensitivity and 99% specificity. In their study, the MD threshold was based on H&E staining, whereas in our investigation, we employed SOX10 staining as the ground truth. We acknowledge that this numeric threshold may not directly translate to our study, as SOX10 staining has been shown to identify more melanocytes compared to H&E staining [12]. The exact cutoff value can be subject to change; nonetheless, we assert that this study underscores the capabilities of the AI model and its potential, notwithstanding the possibility of a different MD cutoff for determining recurrence risk.emerging in future investigations.

Deep learning models have shown potential in melanoma diagnostics. Kucharski et al. performed patch-level segmentation for detection of melanocytic nests and nevus cells on histopathological images using autoencoders [23]. Another study used weakly-supervised training for identifying melanocytic proliferations on histopathology images [24]. Previous studies using AI in the assessment of histopathological resection margins are still rare. Previously, a deep learning model was used to assess the resection margins in basal cell carcinoma during Mohs micrographic surgery showing it to be a feasible option to improve the clinical workflow and reduce costs in histopathological analysis which is in line with the findings in our study [17].

Even though the AI model showed higher sensitivity compared to the pathologists, the specificity was lower. The algorithm tended to identify hyperchromatic keratinocytic nuclei with a surrounding halo as melanocytes contributing to this. Another factor that can cause problems for the observer and the algorithm is the presence of obscuring melanin pigmentation which can be abundantly present in keratinocytes on sun damaged skin). We believe that a larger sample size could further improve the performance of the model in the future. Furthermore, the model could be further adjusted as less sensitive and more specific. However, in this study the current model with high sensitivity was shown to aid the pathologists and improve their performance. The annotation process could also be further optimized if serial sectioned WSI with more extensive IHC were employed in the training set. The material was retrospectively collected from the archives, and additional staining was only performed on the test set due to economic constraints. Using overlayed “virtual slides” of H&E and SOX10 could also help the annotating dermatopathologist to more precisely annotate each single melanocyte [25]. A further improvement to decrease the workload would have been to generate the annotations AI-assisted with help of IHC [26].

The reference standard was based on one pathologist's interpretation of IHC and H&E slides. A consensus of several pathologist interpretations could have made the reference standard more reliable. This could overcome some of the issues we observed with false positivity which could have been a consequence of background being mistaken for a melanocyte. Another drawback lies in the use of slides from a single pathology department and using a single scanning device limiting external validity. Overall, identifying and annotating melanocytic proliferations in WSI poses significant challenges due to the varied sizes and shapes of melanocytic nests, the small size of some melanocytes and the pleomorphism of others showing great heterogeneity and sometimes a much larger nucleus and abundant cytoplasm [27]. Furthermore, a typical LM WSI may contain from a few to several hundreds of nests and several hundreds to thousands of single melanocytes, depending on the case, which resulted in highly labor-intensive annotation processes. Annotating requires the expertise of an experienced dermatopathologist, rendering the collection of an adequate dataset for model training both time-consuming and costly. These obstacles collectively hinder the creation of large, high-quality datasets suitable for training deep learning algorithms. As a future consideration it would be interesting to apply weakly or unsupervised learning methods for assessment of the resection margins.

AI technology may contribute to standardizing outcome predictions and minimizing interobserver variability. Moreover, the consistent performance of the AI model across all times of the day eliminates the impact of fatigue on diagnostic accuracy. However, prior to implementation in real-life scenarios, further validation in larger datasets and across materials from various laboratories scanned with different scanners and stained with different H&E-staining protocols is essential.

## Conclusions

To conclude, the deep learning AI model showed dermatopathologist-level accuracy in the detection of high-risk LM resection margins on routine H&E-stained slides. The use of such a tool could possibly reduce the need for IHC and assist pathologists in their assessment of LM margins.

## Supplementary Information

Supplementary Material 1.

## Data Availability

No datasets were generated or analysed during the current study.

## References

[1] Bosbous MW, Dzwierzynski WW, Neuburg M. Staged excision of lentigo maligna and lentigo maligna melanoma: a 10-year experience. Plast Reconstr Surg. 2009;124:1947–55. 19952650 10.1097/PRS.0b013e3181bcf002

[2] Ungureanu L, Vasilovici AF, Trufin II, Apostu AP, Halmágyi SR. Lentigo maligna treatment-an update. J Clin Med. 2024;13(9):2527. 10.3390/jcm13092527. Published 25 Apr 2024. 38731056 10.3390/jcm13092527 PMC11084749

[3] Hendi A, Wada DA, Jacobs MA, et al. Melanocytes in nonlesional sun exposed skin: a multicenter comparative study. J Am Acad Dermatol. 2011;65:1186–93. 21684036 10.1016/j.jaad.2010.10.039

[4] Barlow JO, Maize J Sr, Lang PG. The density and distribution of melanocytes adjacent to melanoma and nonmelanoma skin cancers. Dermatol Surg. 2007;33:199–207. 17300606 10.1111/j.1524-4725.2006.33039.x

[5] Weyers W, Bonczkowitz M, Weyers I, Bittinger A, Schill WB. Melanoma in situ versus melanocytic hyperplasia in sun-damaged skin. Assessment of the significance of histopathologic criteria for differential diagnosis. Am J Dermatopathol. 1996;18(6):560–6. 8989926 10.1097/00000372-199612000-00002

[6] Dalton SR, Gardner TL, Libow LF. Contiguous lesions in lentigo maligna. J Am Acad Dermatol. 2005;52:859–62. 15858478 10.1016/j.jaad.2004.11.063

[7] Flores S, Luby NJ, Bowen GM. Comparison of melanocyte density counts in topical imiquimod-treated skin surrounding lentigo maligna vs control biopsy specimens. JAMA Dermatol. 2018;154:482–4. 29453870 10.1001/jamadermatol.2017.5632 PMC5876898

[8] Gorman M, Khan MA, Johnson PC, et al. A model for lentigo maligna recurrence using melanocyte count as a predictive marker based upon logistic regression analysis of a blinded retrospective review. J Plast Reconstr Aesthet Surg. 2014;67:1322–32. 24939827 10.1016/j.bjps.2014.05.058

[9] de Wet J, Plessis PJD, Schneider JW. Staged excision of lentigo maligna of the head and neck: assessing surgical excision margins with Melan A, SOX10, and PRAME immunohistochemistry. Am J Dermatopathol. 2023;45(2):107–12. 36669074 10.1097/DAD.0000000000002354

[10] Nybakken GE, Sargen M, Abraham R, et al. MITF accurately highlights epidermal melanocytes in atypical intraepidermal melanocytic proliferations. Am J Dermatopathol. 2013;35:25–9. 22668579 10.1097/DAD.0b013e31825666c3 PMC3581025

[11] Mu EW, Quatrano NA, Yagerman SE, et al. Evaluation of MITF, SOX10, MART-1, and R21 immunostaining for the diagnosis of residual melanoma in situ on chronically sun-damaged skin. Dermatol Surg. 2018;44:933–8. 29419543 10.1097/DSS.0000000000001493

[12] Siarov J, Neittaanmäki N, Mölne J, Gillstedt M, Paoli J. Digital quantification of melanocytic density in resection margins of lentigo maligna using SOX10 versus hematoxylin-eosin staining. Am J Dermatopathol. 2021;43(4):273–7. 32675472 10.1097/DAD.0000000000001749

[13] Acs B, et al. Artificial intelligence as the next step towards precision pathology. J Intern Med. 2020;288(1):62–8. 32128929 10.1111/joim.13030

[14] Grant SR, Andrew TW, Alvarez EV, Huss WJ, Paragh G. Diagnostic and prognostic deep learning applications for histological assessment of cutaneous melanoma. Cancers (Basel). 2022;14(24):6231. Published 17 Dec 2022. 36551716 10.3390/cancers14246231 PMC9776963

[15] Niazi MKK, et al. Digital pathology and artificial intelligence. Lancet Oncol. 2019;20:e253. 31044723 10.1016/S1470-2045(19)30154-8 PMC8711251

[16] Komura D, Ishikawa S. Machine learning approaches for pathologic diagnosis. Virchows Arch. 2019;475:131–8. 31222375 10.1007/s00428-019-02594-w

[17] van Zon MCM, van der Waa JD, Veta M, Krekels GAM. Whole-slide margin control through deep learning in Mohs micrographic surgery for basal cell carcinoma. Exp Dermatol. 2021;30(5):733–8. 33656186 10.1111/exd.14306

[18] Florell SR, Boucher KM, Leachman SA, et al. Histopathologic recognition of involved margins of lentigo maligna excised by staged excision: an interobserver comparison study. Arch Dermatol. 2003;139(5):595–604. 12756096 10.1001/archderm.139.5.595

[19] Mohamed A, Gonzalez RS, Lawson D, Wang J, Cohen C. SOX10 expression in malignant melanoma, carcinoma, and normal tissues. Appl Immunohistochem Mol Morphol. 2013;21(6):506–10. 10.1097/PAI.0b013e318279bc0a. 23197006 10.1097/PAI.0b013e318279bc0a

[20] Bowen AR, Thacker BNP, Goldgar DE, et al. Immunohistochemical staining with Melan-A of uninvolved sun-damaged skin shows features characteristic of lentigo maligna. Dermatol Surg. 2011;37:657–63. 21446989 10.1111/j.1524-4725.2011.01946.x

[21] Gradecki SE, Valdes-Rodriguez R, Wick MR, Gru AA. PRAME immunohistochemistry as an adjunct for diagnosis and histological margin assessment in lentigo maligna. Histopathology. 2021;78(7):1000–8. 10.1111/his.14312. 33280156 10.1111/his.14312

[22] Löper R, Schön MP, Mitteldorf C. Melanocyte density in the diagnosis of melanoma in situ in sun-damaged skin. Am J Dermatopathol. 2024. 10.1097/DAD.0000000000002680. 10.1097/DAD.0000000000002680 38513120

[23] Kucharski D, Kleczek P, Jaworek-Korjakowska J, Dyduch G, Gorgon M. Semisupervised nests of melanocytes segmentation method using convolutional autoencoders. Sensors. 2020;20(6):1546. 32168748 10.3390/s20061546 PMC7146382

[24] Liu K, et al. Learning melanocytic proliferation segmentation in histopathology images from imperfect annotations. In: Paper presented at: 2021 IEEE/CVF conference on Computer Vision and Pattern Recognition Workshops (CVPRW). Nashville; 2021. 10.1109/CVPRW53098.2021.00417.

[25] Jackson CR, Sriharan A, Vaickus LJ. A machine learning algorithm for simulating immunohistochemistry: development of SOX10 virtual IHC and evaluation on primarily melanocytic neoplasms. Mod Pathol. 2020;33(9):1638–48. 32238879 10.1038/s41379-020-0526-z PMC10811656

[26] Nielsen PS, Georgsen JB, Vinding MS, Østergaard LR, Steiniche T. Computer-assisted annotation of digital H&E/SOX10 dual stains generates high-performing convolutional neural network for calculating tumor burden in H&E-stained cutaneous melanoma. Int J Environ Res Public Health. 2022;19:14327. 36361209 10.3390/ijerph192114327 PMC9654525

[27] Reed RJ. The histological variance of malignant melanoma: the interrelationship of histological subtype, neoplastic progression, and biological behavior. Pathology. 1985;17(2):301–12. 4047736 10.3109/00313028509063772

