# Supplementary material for: Utilizing deep learning model for assessing melanocytic density in resection margins of lentigo maligna
Source: Diagn Pathol. 2024 Aug 3;19:106. doi: 10.1186/s13000-024-01532-y (PMC11297622; doi:10.1186/s13000-024-01532-y)

**Additional files**

**Additional figure 1.** The receiver operating characteristic curves and corresponding AUC for the two senior dermatopathologist with and without the aid of AI compared to the AI model (red curve).

**Additional figure 2.** (**a-c**) A test region with true positive AI prediction that was missed by one experienced dermatopathologist but then correctly labeled when aided by the AI. (**d-f**) Another test region with false positive AI prediction that was correctly labeled by the pathologists. (**a,d**) H&E-stained region (**b,e**) H&E-stained region showing green overlaid dots with AI assistance in which the model identified melanocytes and (**c,f**) SOX10-stained region used as ground truth not available for the testers.


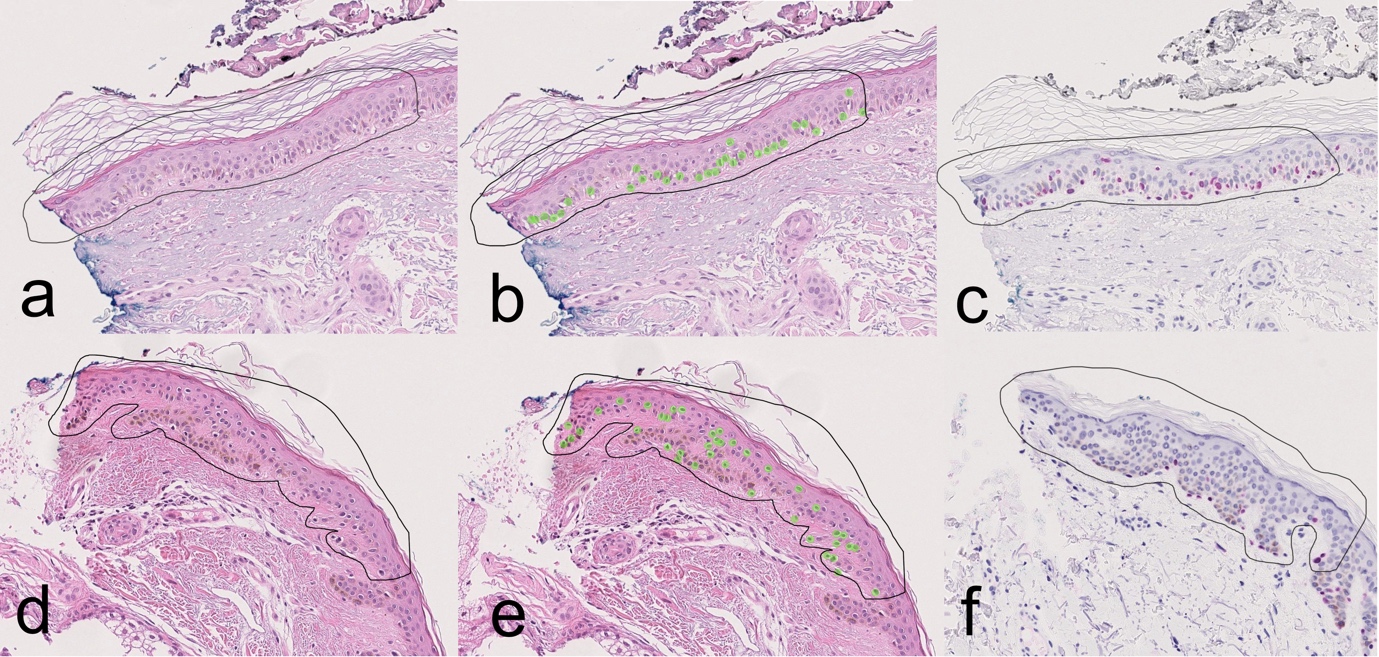

Supplement: Supplementary file 1 — Supplementary Material 1. [file 13000_2024_1532_MOESM1_ESM.docx]
